# Entero-Urethroplasty for Bulbar Urethral Necrosis

**DOI:** 10.7759/cureus.88053

**Published:** 2025-07-16

**Authors:** Muhamed Tajudeen, Sandeep Kumar, Partho Mukherjee, Santosh Kumar

**Affiliations:** 1 Urology, Christian Medical College, Vellore, IND

**Keywords:** bulbar urethra ischemic necrosis, pelvic fracture urethral distraction defect, pelvic fracture urethral injury, reconstructive urology and medical education, urethroplasty complications

## Abstract

Ischemic necrosis of the bulbar urethra after perineal urethroplasty can lead to complex, long-segment strictures. Traditional approaches often involve vascular reconstruction followed by transpubic urethroplasty. A 20-year-old man presented with a posterior urethral injury resulting in urethral distraction following a road traffic accident. He underwent anastomotic urethroplasty 10 months later, which then failed. We employed ascending colon substitution urethroplasty to manage a case of ischemic bulbar necrosis, offering an alternative to tubed flap or staged grafting procedures.

## Introduction

Ischemic bulbar necrosis is a rarely reported complication following progressive perineal urethroplasty. Approximately 9% of patients who undergo surgical repair develop ischemic necrosis of the bulbar urethra [1]. The primary urethral injury at the bulbo-membranous junction, resulting from the pelvic fracture, disrupts the antegrade vascular supply of the bulbar urethra. Ischemic necrosis occurs when the retrograde supply from the dorsal penile artery is also compromised, either due to a congenital defect like hypospadias or, more commonly, iatrogenic injury to the circumflex arteries or dorsal penile arteries during surgical repair, often resulting from excessive urethral mobilization or inferior pubectomy [2]. Rarely, ischemic necrosis of the bulbar urethra can result from the primary pelvic fracture associated with vascular injury itself [1]. Here, we report a case of ischemic necrosis of the bulbar urethra in a young male with pelvic fracture urethral injury following progressive perineal urethroplasty, managed by using ascending colon substitution urethroplasty.

## Case presentation

**Figure 1:**
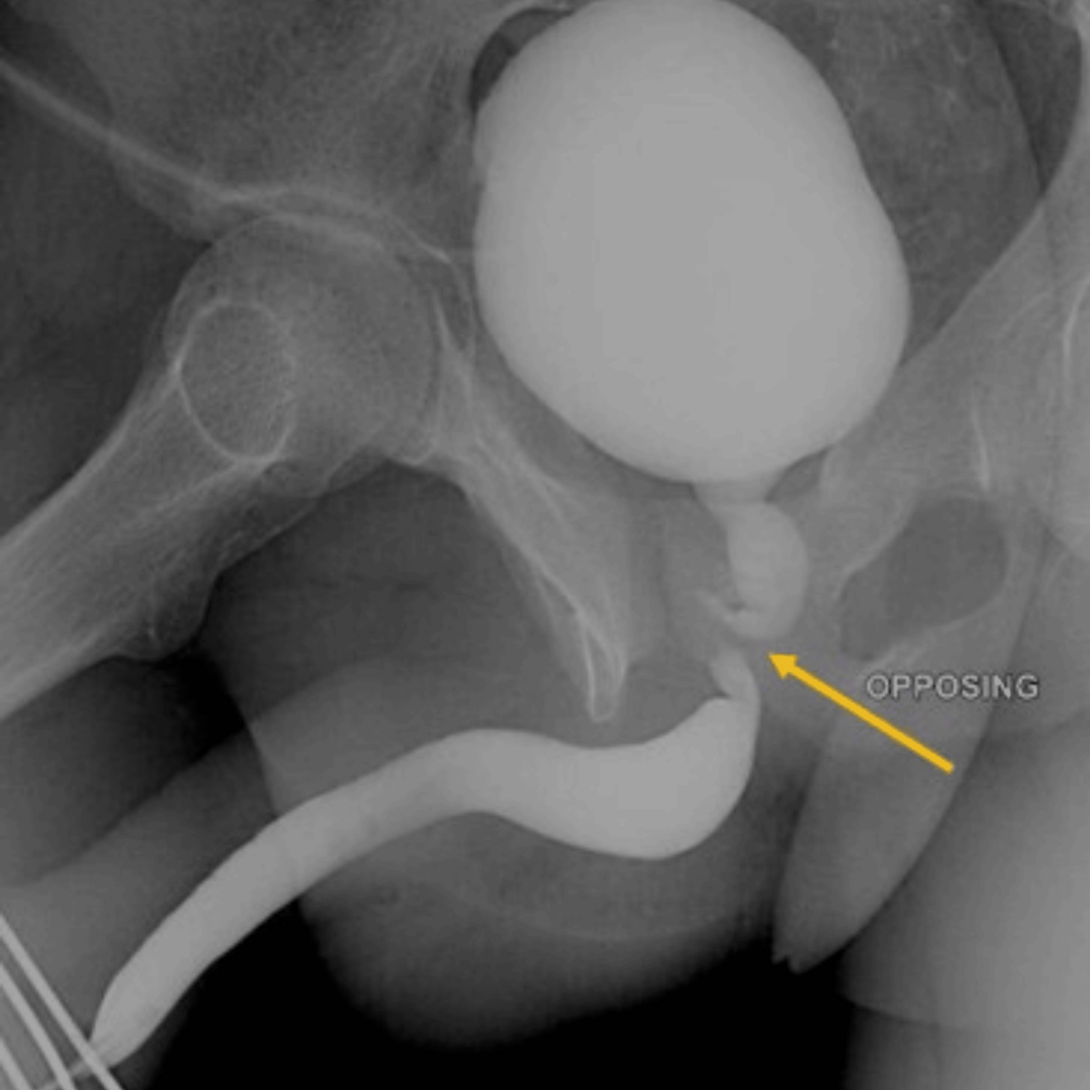
MCU showing posterior urethral distraction defect at the bulbo-membranous junction (yellow arrow). MCU: Micturating cystourethrogram

A 20-year-old man presented with a posterior urethral injury resulting in urethral distraction following a road traffic accident (Figure 1).

**Figure 2:**
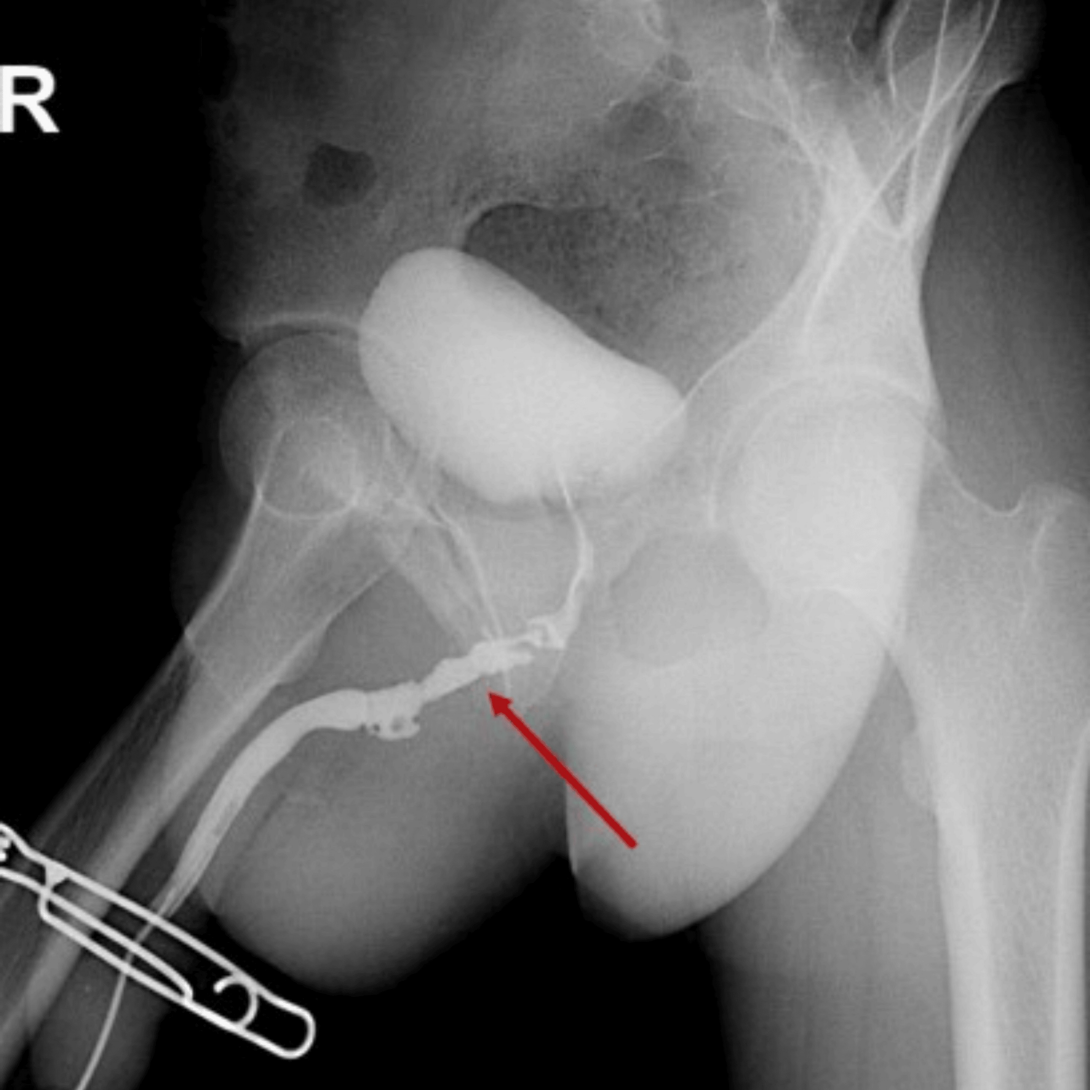
MCU showed a focal narrowing in the proximal bulbar region with saw-tooth appearance of the rest of the bulbar urethra (red arrow). MCU: Micturating cystourethrogram

Initially managed by a suprapubic catheter. The pre-trauma erectile hardness score was four, and the post-trauma erectile hardness score was three. He underwent anastomotic urethroplasty 10 months later, which involved mainly right-sided inferior pubectomy. His post-surgery erectile hardness score was two. He failed to void post-catheter removal. He did not have any perineal pain, perineal swelling, or fever. His initial micturating cystourethrogram (MCU) showed serrated urethral borders with irregular luminal narrowing, indicating loss of urethral wall integrity of the bulbar urethra, classically described as a saw tooth appearance (Figure 2).

**Figure 3:**
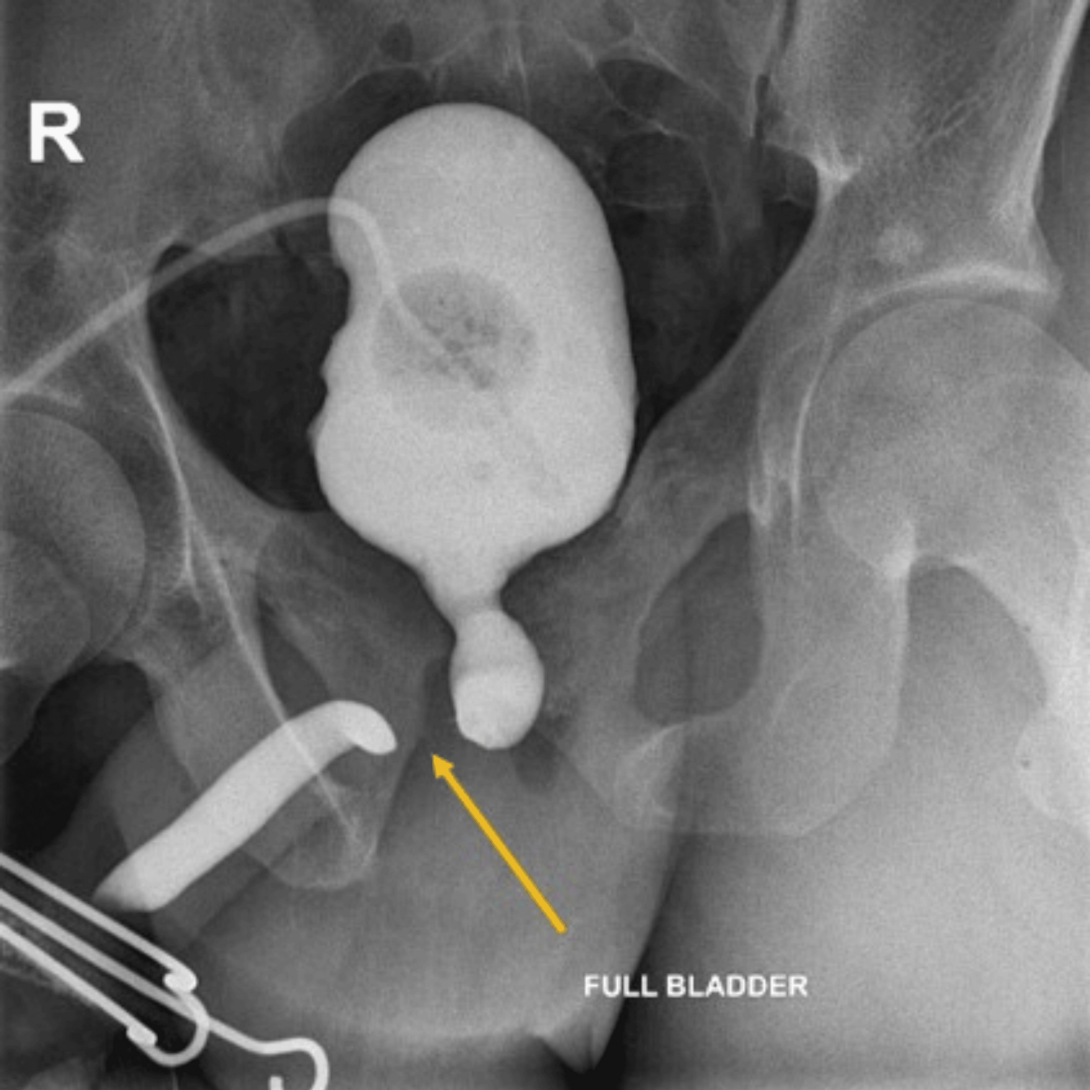
MCU showed complete necrosis of the bulbar urethra (yellow arrow). MCU: Micturating cystourethrogram

Since his erectile function also decreased further after the first surgery, a penile Doppler and subsequent selective pudendal angiography were done, which confirmed that the velocities were suboptimal in both the cavernosal arteries, and there was a complete cut-off of the right dorsal penile artery at the level of inferior pubectomy, and only the left dorsal penile artery was patent. A repeat MCU at 14 months confirmed that there was complete bulbar necrosis (Figure 3).

**Figure 4:**
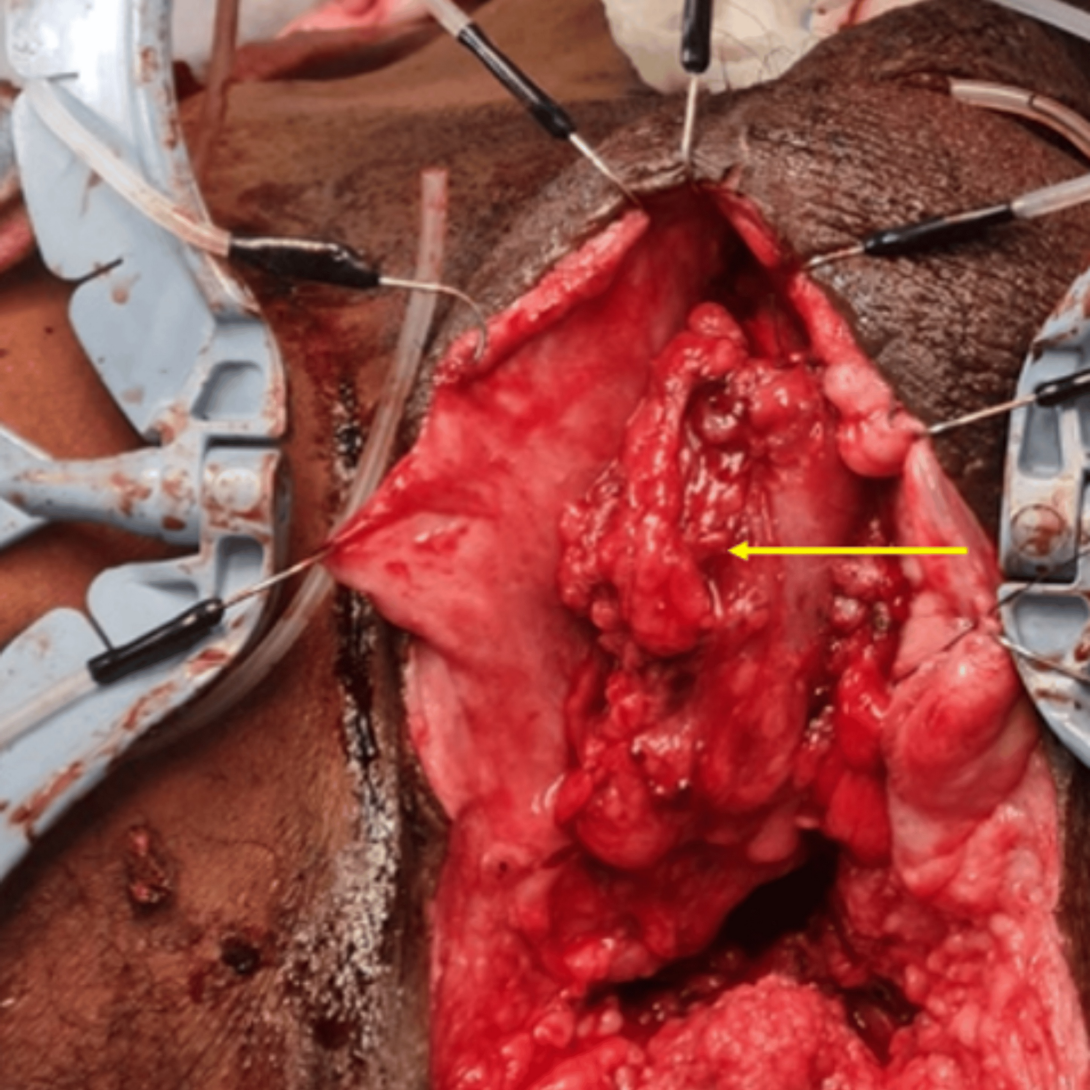
Intraoperative image of entero-urethroplasty with ascending colon (yellow arrow).

He underwent entero-urethroplasty with intraoperative team assistance. The diseased segment of the urethra was excised, and the proximal and distal urethral ends were mobilized and prepared for anastomosis with the selected bowel segment. A vascularized segment of the ascending colon based on the ileocolic artery was harvested, following which an ileocolic anastomosis was performed to maintain bowel continuity. To facilitate the transposition of the bowel into the perineum, the inner plate of the symphysis pubis, along with the intervening cartilage, was excised, thereby creating an adequate subpubic tunnel. A 10 cm segment of the colon, approximately 30 Fr in diameter, was then tunneled into the perineum and positioned between the prepared urethral stumps. The urethro-colonic anastomosis was performed at both ends using interrupted 4-0 polydioxanone (PDS) sutures, with eight sutures placed at each anastomotic site over a 16 Fr Silastic urethral catheter to ensure patency and alignment (Figure 4). 

## Discussion

The bulbar urethra receives a dual blood supply: antegrade flow from the bulbourethral arteries and retrograde perfusion from the dorsal penile arteries [2]. In patients with pelvic fracture urethral injuries (PFUIs), the antegrade blood flow is often compromised. However, the retrograde supply usually remains intact and sufficient to preserve tissue viability. On rare occasions, additional surgical trauma or extensive injury disrupts the retrograde vascular pathway as well, resulting in complete ischemic damage to the bulbar urethra, termed bulbar urethral ischemic necrosis. In such cases, both antegrade and retrograde arterial inflows are compromised, leading to obliterative necrosis of the bulbar segment. A large series of over 1700 patients with PFUI reported only three instances of bulbar urethral ischemic necrosis attributable to the initial trauma, underscoring its rarity [1].

The presence of post-traumatic erectile dysfunction in these patients may indicate underlying vascular compromise of the distal urethra. In such scenarios, preoperative assessment using penile Doppler ultrasound is warranted. Vascular reconstruction may be beneficial before progressive perineal urethroplasty, especially in patients with marginal retrograde flow, to reduce the risk of iatrogenic ischemia during mobilization [1]. However, patients who present with primary ischemic necrosis of the bulbar urethra, as seen in rare cases of direct traumatic vascular disruption, are unlikely to benefit from revascularization procedures due to the extensive devascularization. In these cases, the urethral defect is often too long for primary anastomosis, even with pubectomy, necessitating the use of interposition techniques using flaps or grafts.

In uncircumcised patients, a preputial tubed flap is often the preferred reconstructive option, as it can be harvested with a reliable vascular pedicle based on the dartos fascia. This technique enables single-stage reconstruction while preserving native tissue vascularity [1]. Alternatively, combined techniques employing dorsal onlay grafts and ventral flaps have been described to achieve lumen patency in such complex reconstructions [3]. In circumcised patients, a two-stage urethroplasty with an initial perineal urethrostomy and dorsal grafting followed by tubularization is typically favored. More extensive options, such as the gracilis muscle or radial forearm free flaps, may be considered in selected cases with inadequate local tissue [4].

In our case, we employed ascending colon substitution urethroplasty, which has been previously described as a salvage procedure for bulbourethral strictures [5]. This technique, though rarely used, offers a feasible reconstructive alternative when conventional options are inadequate or unviable. The ascending colon, with its robust blood supply and pliability, can serve as an effective interposition graft, particularly in long-segment urethral defects. This case underscores the potential of colonic substitution urethroplasty in extreme cases of ischemic urethral loss, expanding the reconstructive armamentarium for complex anterior urethral strictures and highlighting a viable pathway for restoring urethral continuity and function in anatomically and surgically challenging settings.

## Conclusions

Bulbar urethral ischemic necrosis is a rare but difficult complication to deal with. Options to manage bulbar urethral necrosis are limited, requiring reconstructive expertise. Often warrants a reconstruction with flaps or grafts to bridge the defect. In uncircumcised patients, a preputial tubed flap is the preferred method. Ascending colon substitution urethroplasty can be a viable management option for complex cases of ischemic necrosis to improve postoperative outcomes.
